# Altered Function of the DnaJ Family Cochaperone DNJ-17 Modulates Locomotor Circuit Activity in a *Caenorhabditis elegans* Seizure Model

**DOI:** 10.1534/g3.116.028928

**Published:** 2016-05-16

**Authors:** Seika Takayanagi-Kiya, Yishi Jin

**Affiliations:** * Section of Neurobiology, Division of Biological Sciences, University of California, San Diego, La Jolla, California, 92093; † Howard Hughes Medical Institute, University of California, San Diego, La Jolla, California, 92093

**Keywords:** GABA, glutamic acid decarboxylase, acetylcholine receptor, excitation, inhibition balance

## Abstract

The highly conserved cochaperone DnaJ/Hsp40 family proteins are known to interact with molecular chaperone Hsp70, and can regulate many cellular processes including protein folding, translocation, and degradation. In studies of *Caenorhabditis elegans* locomotion mutants, we identified a gain-of-function (gf) mutation in *dnj-17* closely linked to the widely used *e156* null allele of *C. elegans* GAD (glutamic acid decarboxylase) *unc-25*. *dnj-17* encodes a DnaJ protein orthologous to human DNAJA5. In *C. elegans* DNJ-17 is a cytosolic protein and is broadly expressed in many tissues. *dnj-17(gf)* causes a single amino acid substitution in a conserved domain, and behaves as a hypermorphic mutation. The effect of this *dnj-17(gf)* is most prominent in mutants lacking GABA synaptic transmission. In a seizure model caused by a mutation in the ionotropic acetylcholine receptor *acr-2(gf)*, *dnj-17(gf)* exacerbates the convulsion phenotype in conjunction with absence of GABA. Null mutants of *dnj-17* show mild resistance to aldicarb, while *dnj-17(gf)* is hypersensitive. These results highlight the importance of DnaJ proteins in regulation of *C. elegans* locomotor circuit, and provide insights into the in vivo roles of DnaJ proteins in humans.

Cells have molecular mechanisms to protect themselves from the stress caused by misfolded or aggregated proteins. DnaJ/Hsp40 family proteins are highly conserved through evolution and act as cochaperones by interacting with and activating the ATPase activity of Hsp70 chaperone proteins (Ohtsuka and Suzuki 2000; Qiu *et al.* 2006). Together, Hsp40 and Hsp70 help folding of nascent proteins and refolding and degradation of misfolded proteins.

Accumulation of protein aggregation underlies various human diseases including neurodegenerative diseases such as Parkinson’s disease and Huntington’s disease (Muchowski and Wacker 2005; Sherman and Goldberg 2001). Mutations in DnaJ/Hsp40 proteins have been associated with such diseases, suggesting the importance of cochaperones in cellular protein homeostasis (Blumen *et al.* 2012; Borrell-Pagès *et al.* 2006; Trinh and Farrer 2013). In *C. elegans*, overexpression of polyglutamine repeats in muscles or neurons causes formation of protein aggregation in an age-dependent manner (Brignull *et al.* 2006a,b), similar to that observed in human polyglutamine diseases (Sakahira *et al.* 2002). In addition, excess excitatory neuronal signaling at the neuromuscular junction causes locomotion defects and increased protein aggregation in muscles in a *C. elegans* polyglutamine disease model, suggesting that neuronal activity can affect protein homeostasis in other tissues (Garcia *et al.* 2007).

Functions of neural circuits depend critically on balanced activity between excitatory and inhibitory transmission. In *C. elegans*, locomotion is controlled by the coordinated activities of excitatory cholinergic and inhibitory GABAergic motor neurons (Von Stetina *et al.* 2006). GABA plays crucial roles in the nervous system of both vertebrates and invertebrates. In *C. elegans*, mutants affecting GABA transmission were isolated from forward genetic screens for locomotor defects (Brenner 1974; Jin *et al.* 1999; McIntire *et al.* 1993). The *C. elegans* genes required for GABA neurotransmission including *unc-25/GAD* (Jin *et al.* 1999), *unc-47/VGAT* (McIntire *et al.* 1997), and *unc-49/GABA_A_R* (Bamber *et al.* 1999; Richmond and Jorgensen 1999) are highly conserved among animals. Analysis of *unc-25/GAD* mutants has revealed that the canonical reference allele *unc-25(e156)* causes a premature termination codon (Trp383amber) in the enzymatic domain; *e156* mutants completely lack GABA immunoreactivity and have been widely used as representative of complete loss of GABA function.

The nicotinic acetylcholine receptor subunit *acr-2* is expressed in the cholinergic motor neurons. A gain-of-function mutation of *acr-2* causes increased cholinergic motor neuron activity accompanied by decreased GABAergic motor neuron activity, generating excitation–inhibition (E/I) imbalance in locomotor circuit. *acr-2(gf)* animals exhibit a characteristic repetitive convulsion behavior, the frequency of which provides a quantitative measure of E/I imbalance (Jospin *et al.* 2009; Stawicki *et al.* 2013).

Through studying the effects of defective GABAergic transmission on *acr-2(gf)* animals, we unexpectedly found a gain-of-function mutation in a cochaperone protein *dnj-17* to be present in the widely used strain CB156 *unc-25(e156)*. We show that the DNJ-17 gain-of-function mutation behaves in a hypermorphic manner, and exacerbates excitation–inhibition imbalance in *acr-2(gf)*. Null mutations of *dnj-17* exhibit mild resistance to aldicarb, suggesting a role in modulating neurotransmission. Homologs of DNJ-17 include human DNAJA5, which is expressed in the brain and other tissues. Our findings provide insights into the *in vivo* function of these cochaperone proteins.

## Materials and Methods

### Strains

*C. elegans* strains were kept at 22.5° according to standard procedures. Supplemental Material, Table S1 lists strain information with alleles and transgenes. Galaxy platform (Giardine *et al.* 2005) and CloudMap workflows (Minevich *et al.* 2012) were used to analyze the whole-genome sequence data of MT6648 *unc-25(e156) dnj-17(ju1162)III*; *acr-2(n2420)X* and CZ19995 *unc-25(e156) dnj-17(ju1162)III*; *acr-2(n2420)X*, obtained by Beijing Genomics Institute (Shenzhen, China). Subsequent analyses based on chromosomal linkage and recombination mapping identified the *ju1162* missense mutation in *dnj-17*. We verified the presence of *dnj-17(ju1162)* in CB156, and generated CZ22168 *unc-25(e156)* that lacks *dnj-17(ju1162)* through multistep recombination as follows: We verified that the Caenorhabditis Genetics Center (CGC) strain SP1104 *unc-25(e156) bli-5(e518)III* is wild type for *dnj-17*. We outcrossed SP1104 to N2, and isolated recombinant animals that showed *unc-25(0)* shrinker phenotype without blister phenotype. We performed genotyping on isogenic strains of the recombinants, and confirmed the presence of *unc-25(e156)* and the loss of *bli-5(e518)*. In this process, we also found SP1104 has another mutation linked to chromosome III that caused egg-laying defects. Through further outcrossing to N2, we reisolated *unc-25(e156)* based on behavior and genotyping and established strain CZ22168 which does not exhibit the egg-laying defects. Primers used for PCR and genotyping were as follows: YJ10801 CCGTAGAAACCATTCACAGTTTGC and YJ10802 CTATGAAATGCCATTACGAAGTGCTC for *dnj-17(ju1162)*, YJ11985 CATTGGCGCAGACTATTGCTTC and YJ11986 AATTGCTCACCGAAACTCACATTCT for *unc-25(e156*), YJ10799 TACTTGGTATCCAGCTCCTTCC and YJ10800 ATTATTTGGACAGTTTAGCCCACC for *bli-5(e518)*. The information on the alleles and CB156 is deposited in CGC and Wormbase. Several researchers noted that *unc-25(e156)* appeared to behave differently from other *unc-25* alleles or GABA mutants, in a number of behavioral and pharmacological assays (C. Bargmann, E. Jorgensen, S. Chalasani, J. Kaplan, personal communications). For future experiments on *unc-25* mutants, we recommend CZ22168, as well as other *unc-25* alleles.

### Molecular biology and transgenes

Molecular biology was performed following standard methods. Gateway recombination technology (Invitrogen, CA) was used for expression vectors. Table S2 describes the details of constructs generated in this study. We amplified 3.5 kb genomic sequences of *dnj-17* with 0.9 kb 5′ upstream sequences to 0.1 kb 3′ downstream region using the following primers: YJ11121 AAACTCCATCAACCTGACTTCCCTG and YJ11122 TTGCCCATTATTCTTCCCGAAAC. To determine the gene structure of *dnj-17*, we isolated mRNAs from mixed-stage animals of N2 wild type and CB156 *unc-25(e156) dnj-17(ju1162)* using Trizol (ThermoFisher Scientific). Complementary DNA (cDNA) synthesis was performed using SuperScript III (ThermoFisher Scientific), with random primers according to the manufacturers’ instructions. We performed RT-PCR using SL1 primer GTTTAATTACCCAAGTTTGAG and a reverse primer p3 GCGACCAGATTCCTAATTTGCTCGTTC designed on the junction of exon 3 and exon 4 to determine the first exon of *dnj-17* mRNA, and p2 ATGAAATGCCATTACGAAGTGCTC and p4 AATGTTTCACCAATCCTCATCATCC primers designed on the first and sixth exon to verify the coding sequence. Sequences of all clones were verified by Sanger sequencing. Protein domain analysis was performed using NCBI domain database (Marchler-Bauer *et al.* 2015) and Treefam (Li *et al.* 2006).

### Generation of deletion alleles of dnj-17 by CRISPR-Cas9 editing

*dnj-17(ju1239)* and *dnj-17(ju1276)* deletion alleles were generated by CRISPR-Cas9 editing in the germline, using modifications of previously described methods (Dickinson *et al.* 2013) (Z. Wang and Y.J., unpublished data). Briefly, adult animals were injected with the Cas9-sgRNA expression constructs (pCZGY2647 and pCZGY2646, made from pDD162 with sgRNA) and *Pmyo-2-mCherry* as a co-injection marker. F1 animals expressing mCherry in pharynx were isolated, allowed to lay eggs, and then genotyped for *dnj-17* to detect deletions. The F2 progeny of F1 animals with deletions were isolated to establish strains containing *dnj-17* deletion. sgRNA sequences used to target *dnj-17* are the following: ACAGAAAACTAGCGCTCAAA and GAGTTTGGCGACAAGGATAC. *ju1239* was generated following microinjection into N2 animals. *ju1276* was generated following microinjection into CB156 *unc-25(e156)* animals.

To analyze the temperature effects on *dnj-17(ju1239)*, we examined the growth and locomotion of N2 and CZ21429 *dnj-17(ju1239)* under different temperature conditions. Briefly, 10 gravid adults of each strain were allowed to lay eggs for 6 hr at 22.5°. Then, adult animals were removed, and the plates with embryos were kept under 15°, 22.5°, or 25°. Hatched progeny were kept under the same temperature, and their growth and general locomotion were visually scored once within 16−24 hr. When the progeny reached L4 stage, animals from each condition were placed onto individual plates. Number of eggs laid by each animal was scored to compare the brood size. The experiment was repeated twice.

### Generation of single-copy inserted strains

Single-copy insertion transgenes of *Pdnj-17-dnj-17(+)* and *Pdnj-17-dnj-17(ju1162)* were generated at Chromosome II site ttTi5605 using modified vectors (Z. Wang and Y.J., unpublished data). Briefly, N2 young adult animals were injected with the following constructs: a construct (pCZGY3031 or pCZGY3032) containing *dnj-17* sequence with ttTi5605 homology arms and a copy of hygromycin resistance gene, a construct (pDD122) which drives expression of Cas9 and sgRNA targeting ttTi5605 in the germline, and a *Pmyo-2-mCherry* fluorescent co-injection marker. F2 animals were selected for the resistance to hygromycin. Single-copy insertion lines were confirmed by PCR using primers designed outside of the homology arms. Loss of extrachromosomal array was confirmed by PCR and the loss of co-injection marker. Each insertion line was outcrossed twice before being used in experiments.

### Quantification of convulsion behavior

Scoring of convulsions was performed as previously described (Stawicki *et al.* 2013). Briefly, L4 larvae were transferred to nematode growth medium (NGM) plates seeded with *Escherichia coli* OP50. On the following day, young adults were transferred to fresh plates with OP50 and visually scored for convulsion behavior under a dissecting microscope. The observer was blinded to the genotype of the animals tested. A convulsion event was defined as a shortening of the animal’s body length. The assay was repeated at least twice per genotype in two different generations. Two independent transgenic lines were used for each construct.

### Aldicarb assay

One day before the experiment, L4 animals were transferred to fresh plates seeded with OP50. On the next day, 10 animals were transferred to an NGM plate with 500 µM aldicarb. Animal behavior was scored every 30 min. Animals were scored paralyzed when they did not move for more than 5 sec in response to touch stimulus.

### Confocal microscopy

L4 animals were imaged using a Zeiss LSM 710 confocal microscope (63× objective). Animals were immobilized by 1 mM levamisole and placed on 4% agar pads. Images are maximum-intensity projections of z stacks obtained at 1 µm intervals. ImageJ was used to process the images obtained.

### Data availability

Strains and constructs are described in Table S1 and Table S2 respectively, and are available upon request.

## Results and Discussion

### Identification of a missense mutation in dnj-17 in unc-25(e156) strains

**Figure 1:**
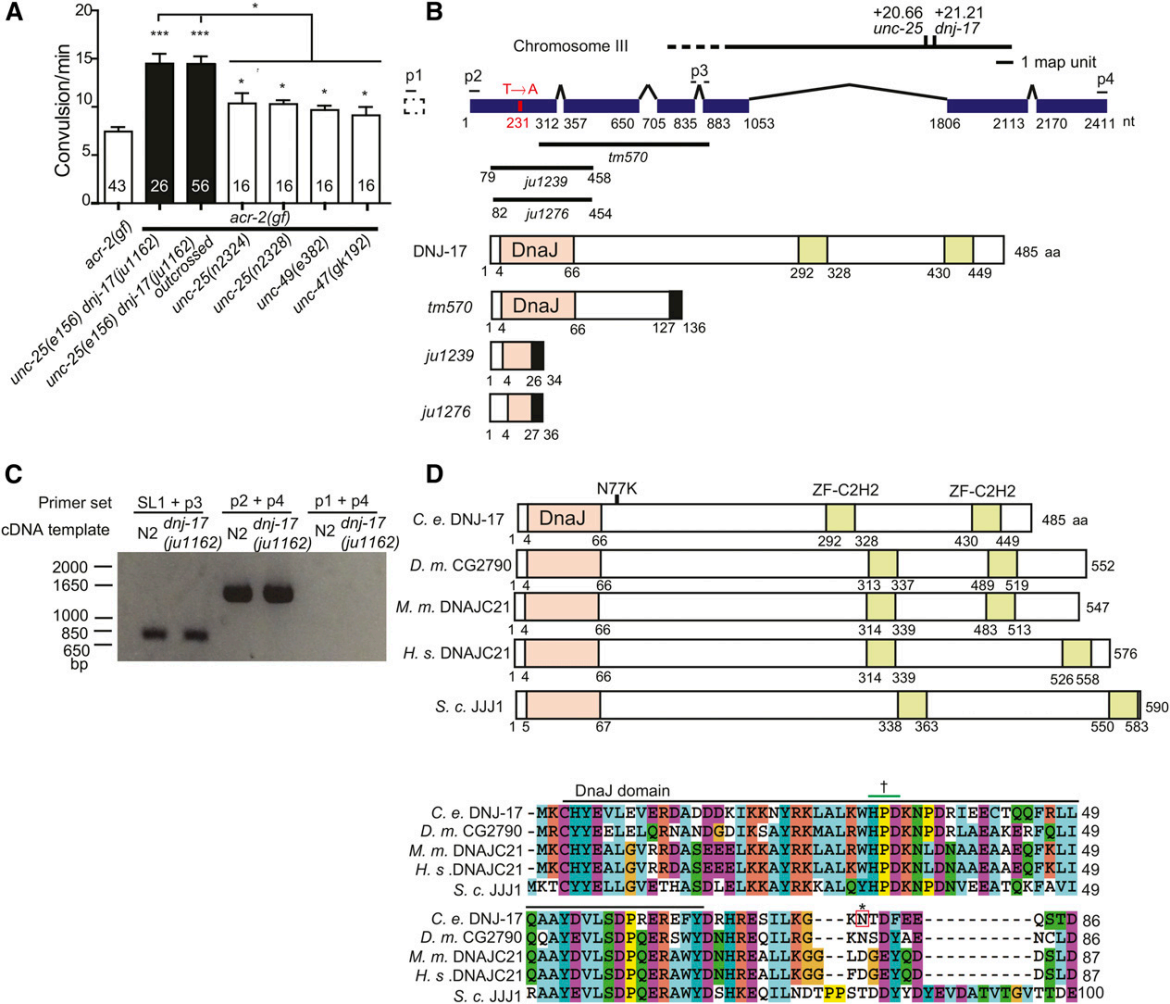
Single amino acid substitution in DNJ-17 in the background of *unc-25(e156)* causes increase of *acr-2(gf)* convulsions. (A) Quantification of convulsion frequency of strains with mutations in genes required for GABA transmission. Note the higher convulsion frequency of the animals with *unc-25(e156) dnj-17(ju1162)*. Statistics, one way ANOVA followed by Bonferroni’s *post hoc* test. * *P* < 0.05, *** *P* < 0.001. Error bars indicate SEM. Numbers in the column indicate sample sizes. (B) Upper panel shows *dnj-17* gene structure, with the location of the *ju1162* T to A nucleotide change, deletion mutations, and primers (p1, p2: forward primer, p3, p4: reverse primer) designed for cDNA amplification. Lower panel shows predicted DNJ-17 protein in wild type and in deletion mutants. All three mutations cause frameshift and produce premature stop codons. Note that *ju1239* and *ju1276* remove the highly conserved DnaJ domain. Black fills designate frame-shifted regions. (C) Gel electrophoresis of the cDNA fragments amplified by PCR using the designated primers (shown in B). SL1 with p3 primer amplified cDNA fragment including the start codon. (D) Upper panel shows DNJ-17 family protein structure. *D. m*.: *Drosophila melanogaster*, *M. m*.: *Mus musculus*, *H.s*.: *Homo sapiens*. *S.c*.: *Saccharomyces cerevisiae*. Lower panel shows amino acid sequence alignment around the DnaJ domain. *, position of N to K mutation in *ju1162*; †, HPD motif, highly conserved among the J domain and required for the activation of Hsp70 (Tsai and Douglas 1996; Meyer *et al.* 2007).

*acr-2(n2420gf)* animals show spontaneous convulsion behavior, due to increased cholinergic excitation and reduced GABAergic inhibition (Jospin *et al.* 2009). We wanted to further examine the effects of GABAergic transmission on the convulsive behavior of *acr-2(gf)* animals. We generated double mutants of *acr-2(gf)* with genes essential for GABA signaling, using canonical or null alleles of *unc-25/GAD*, *unc-47/VGAT*, *unc-49/GABAR*. We used three null mutations of *unc-25: e156*, *n2324*, and *n2328*, which cause amber stop codons at Trp383, Trp291, and Glu486, respectively, and which are all predicted to encode truncated proteins that lack the cofactor binding site and enzymatic activity site at the C-terminus. All double mutants showed increased convulsion frequency compared to *acr-2(gf)* single mutants (Figure 1A). While *unc-25(n2324)* and *unc-25(n2328)* enhanced *acr-2(gf)* behavioral defects to similar degrees as *unc-47(gk192)* and *unc-49(e382)*, *unc-25(e156)* increased the convulsion frequency significantly more than these four mutations. Further outcrossing of *unc-25(e156)*; *acr-2(gf)* (MT6648) did not eliminate this enhancement. We thus hypothesized that the ancestral CB156 strain may contain additional modifier mutation(s) linked to *unc-25(e156)*.

We performed whole-genome sequencing analysis of MT6648 and of an outcrossed strain CZ19995 *unc-25(e156)*; *acr-2(gf)*. Following chromosomal linkage mapping, we identified a single nucleotide transversion from thymine to adenine in the coding sequence of the gene *dnj-17*, approximately 0.5 map units right of *unc-25* on chromosome III (Figure 1B), and hereafter referred to as *dnj-17(ju1162)*. The *ju1162* mutation was present in the CGC strain CB156 *unc-25(e156)*, but not in SP1104 *unc-25(e156) bli-5(e518) III*, which was generated in about 1987 through recombination from *trans*-heterozygous animals of *unc-25(e156)* with a chromosome containing *bli-5(e518)* (R. Herman, personal communication). *dnj-17(ju1162)* was also not present in MT5957 *unc-25(n2324) III* and MT5969 *unc-25(n2328) III*. Therefore, *dnj-17(ju1162)* did not arise as a spontaneous mutation in strain passage in our laboratory, but was inherited from the original CB156 stock.

### dnj-17 encodes a homolog of human DNAJA5

Gene structure (Wormbase WS251) showed that *dnj-17* contains seven exons, generating a mature mRNA predicted to encode a protein of 510 amino acids. To verify the *dnj-17* gene structure we performed cDNA analyses using mRNA isolated from N2 and CB156 *unc-25(e156) dnj-17(ju1162)*. RT-PCR analyses using SL1 and gene-specific primers revealed that the 5′ end of *dnj-17* mRNA contained an SL1 leader, but predicted exon 1 was not present in the mature mRNA. We obtained full-length *dnj-17* cDNA and found that DNJ-17 protein consists of 485 amino acids (Figure 1, B and C). From NCBI protein domain analysis, the N-terminus of DNJ-17 has a highly conserved DnaJ domain, known to interact with Hsp70 family proteins, and the C-terminal half contains two C2H2-type zinc finger motifs that have been implicated to be important for polypeptide binding (Banecki *et al.* 1996; Lu and Cyr 1998; Szabo *et al.* 1996). Relatives of DNJ-17 are found widely in eukaryotes, with orthologs named as JJJ1 in yeast, DNAJA5/DNAJC21 in human, DNAJC21 in mouse, and CG2790 in *Drosophila* (Figure 1D). The J domain of DNJ-17 also has a highly conserved HPD motif that is crucial for interaction with Hsp70 proteins (Tsai and Douglas 1996). Yeast JJJ1 activates ATPase activity of Hsp70, and lack of JJJ1 results in cold sensitivity (Meyer *et al.* 2007). On the other hand, functions of the DNJ-17 family proteins in animals remain mostly unknown, though human *DNAJA5* is expressed in several tissues including the brain (Chen *et al.* 2004). We confirmed that cDNAs from CB156 *unc-25(e156) dnj-17(ju1162)* contained a single nucleotide change, which causes Asp77 to Lys amino acid substitution (N77K) in the region immediately adjacent to the DnaJ domain (Figure 1, B and D).

### dnj-17(ju1162) is a gain-of-function mutation

**Figure 2:**
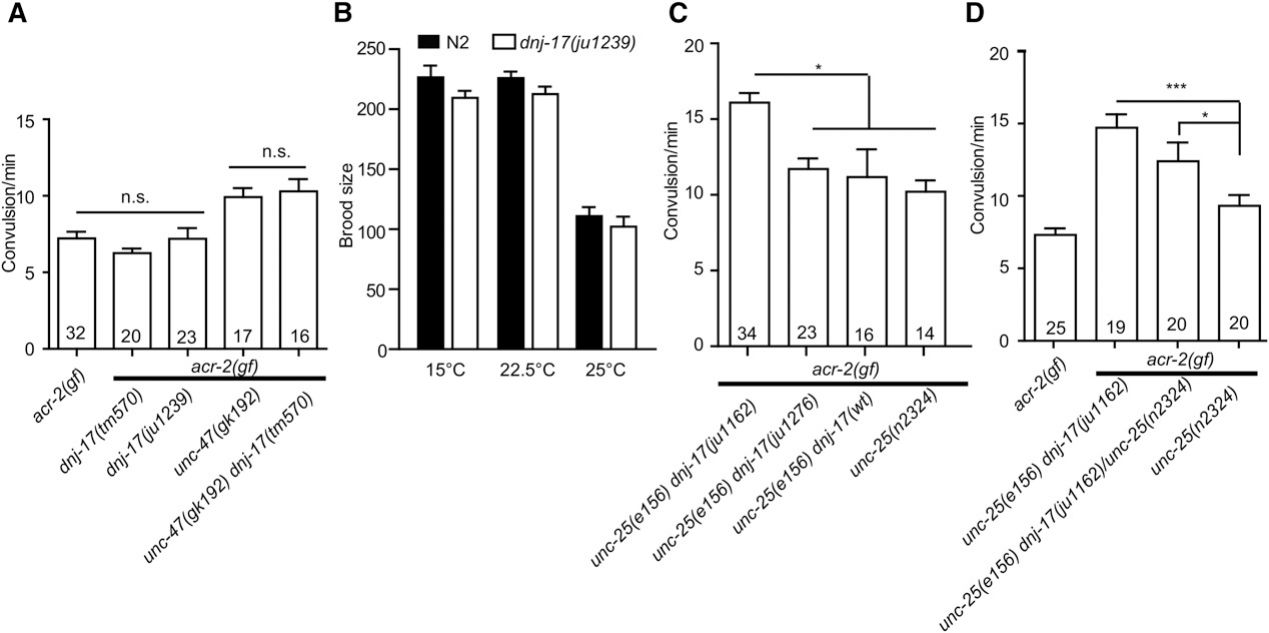
*dnj-17(ju1162)* acts as a gain-of-function mutation. (A,C,D) Quantification of convulsion frequency. (A) Loss-of-function deletion alleles of *dnj-17* do not affect *acr-2(gf)* convulsion frequency. (C) Removal of *dnj-17(ju1162gf)* reduces the convulsion frequency. (D) *dnj-17(ju1162gf)* shows semidominant effects on convulsion frequency. Statistics, one way ANOVA followed by Bonferroni’s *post hoc* test. * *P* < 0.05. Numbers in the column indicate sample sizes. (B) Brood size is not affected by deletion of *dnj-17*. *n* = 6 for each condition. Error bars indicate SEM.

Several lines of evidence support that *ju1162* is a gain-of-function mutation of *dnj-17*. First, we examined the effect of a *dnj-17* deletion allele *dnj-17(tm570)*, which removes the C-terminal half of the protein (Figure 1B). *dnj-17(tm570)* homozygous animals showed normal growth rate, wild-type locomotion, and did not affect convulsion frequency of *acr-2(gf)* (Figure 2A). We also generated *unc-47(gk192) dnj-17(tm570)*; *acr-2(n2420)* triple mutants and found that they resembled *unc-47(gk192)*; *acr-2(n2420)* double mutants in their convulsion frequency. As *dnj-17(tm570)* mutants potentially produce mRNAs encoding a truncated protein with intact DnaJ domain, we next generated a deletion allele targeting the DnaJ domain using CRISPR-Cas9-mediated genome editing technology (Dickinson *et al.* 2013; Friedland *et al.* 2013) (Z. Wang and Y.J., unpublished results). *dnj-17(ju1239)* removes a large portion of the DnaJ domain and is predicted to cause a frameshift and premature stop after amino acid 34 (Figure 1B). Since the null mutation of a yeast protein with J domain, *Jjj1*, was previously reported to cause cold sensitivity in yeast (Meyer *et al.* 2007), we examined the viability and locomotion of *dnj-17(ju1239)* mutants. The mutant animals had similar brood size as wild type under three temperature conditions (Figure 2B). Their growth rate, body shape, and movement were also indistinguishable from wild type. Finally, *dnj-17(ju1239)* did not affect the convulsion frequency of *acr-2(n2420)* (Figure 2A). These observations show that *dnj-17* is a nonessential gene for *C. elegans* development and behavior, and that *dnj-17* loss-of-function does not affect convulsion of *acr-2(gf)* by itself or when GABA transmission is eliminated in *unc-47(null)* animals.

We further examined if removing *dnj-17(ju1162)* from *unc-25(e156)* background would eliminate the increased convulsion frequency phenotype of *acr-2(gf)*. As *dnj-17* is located 0.5 map unit apart from *unc-25*, it is challenging to separate *unc-25(e156)* and *dnj-17(ju1239)* by genetic recombination. We therefore generated another deletion allele in the *unc-25(e156) dnj-17(ju1162)* background using CRISPR editing (Figure 1B). *dnj-17(ju1276)* removed the DnaJ domain and the region including *ju1162(N77K)*, and eliminated the increased convulsion (Figure 2C). Furthermore, through isolation of *unc-25(e156)* recombinants after outcrossing SP1104 *unc-25(e156) dnj-17(+) bli-5(e518)*, we obtained CZ22169 *unc-25(e156) dnj-17(+)*; *acr-2(n2420)*. Animals of genotype *unc-25(e156) dnj-17(ju1276)*; *acr-2(gf)* and *unc-25(e156) dnj-17(+)*; *acr-2(gf)* showed convulsion frequencies lower than *unc-25(e156) dnj-17(ju1162)*; *acr-2(gf)*, and instead resembled *acr-2(gf)* double mutants with *unc-25(n2324)* or with other GABA mutants (Figure 2C). Finally, we also observed that *dnj-17(ju1162)* showed semidominant effects on convulsion frequency in the *acr-2(gf)*; *unc-25(0)* background (Figure 2D). Thus, we conclude that *dnj-17(ju1162)* is a semidominant gain-of-function mutation, designated as *dnj-17(ju1162gf)*.

### DNJ-17 is a cytosolic protein expressed in multiple tissues

**Figure 3:**
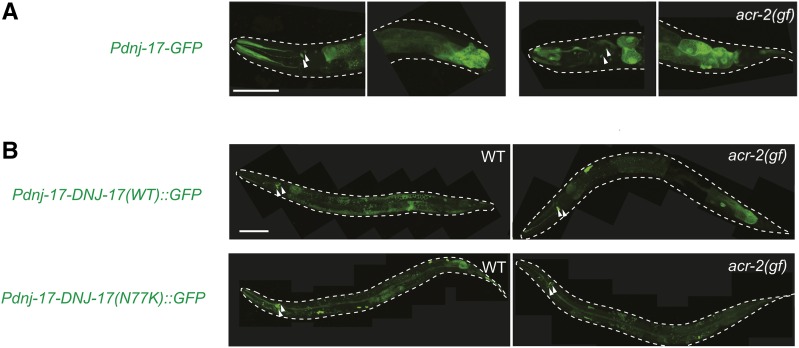
Expression pattern of DNJ-17 is not affected by the N77K mutation or by *acr-2(gf)*. (A) Confocal images of animals expressing *dnj-17* transcriptional reporter. (B) Confocal images of animals expressing DNJ-17 translational reporter. Scale bar: 50 µm. Arrowheads point to expression in cell bodies of the head neurons.

We next analyzed the expression pattern of *dnj-17*. We first generated an extrachromosomal transcriptional green fluorescent protein (GFP) reporter using 0.9 kb promoter region of *dnj-17*. GFP was seen throughout the body with enrichment in the intestine and several cells around the pharynx, and the expression pattern was similar in both wild type and *acr-2(gf)* background (Figure 3A). We then made GFP-fused translational DNJ-17 reporters. GFP signals from *Pdnj-17-dnj-17*::*gfp* localized to the cytosol of head neurons, and in other unidentified cells at lower levels throughout the body (Figure 3B). A weaker but similar pattern was observed in an integrated fosmid expression line which expresses DNJ-17 tagged with C-terminal TY1::EGFP:3xFLAG (not shown) (Zhong *et al.* 2010). Moreover, DNJ-17(N77K)::GFP showed similarly diffused expression. Both DNJ-17(+)::GFP and DNJ-17(N77K) expression patterns were similar in wild type and in *acr-2(gf)* background, suggesting that the presence of *acr-2(gf)* does not largely affect the localization of DNJ-17.

### DNJ-17(N77K) behaves as a hypermorph

**Figure 4:**
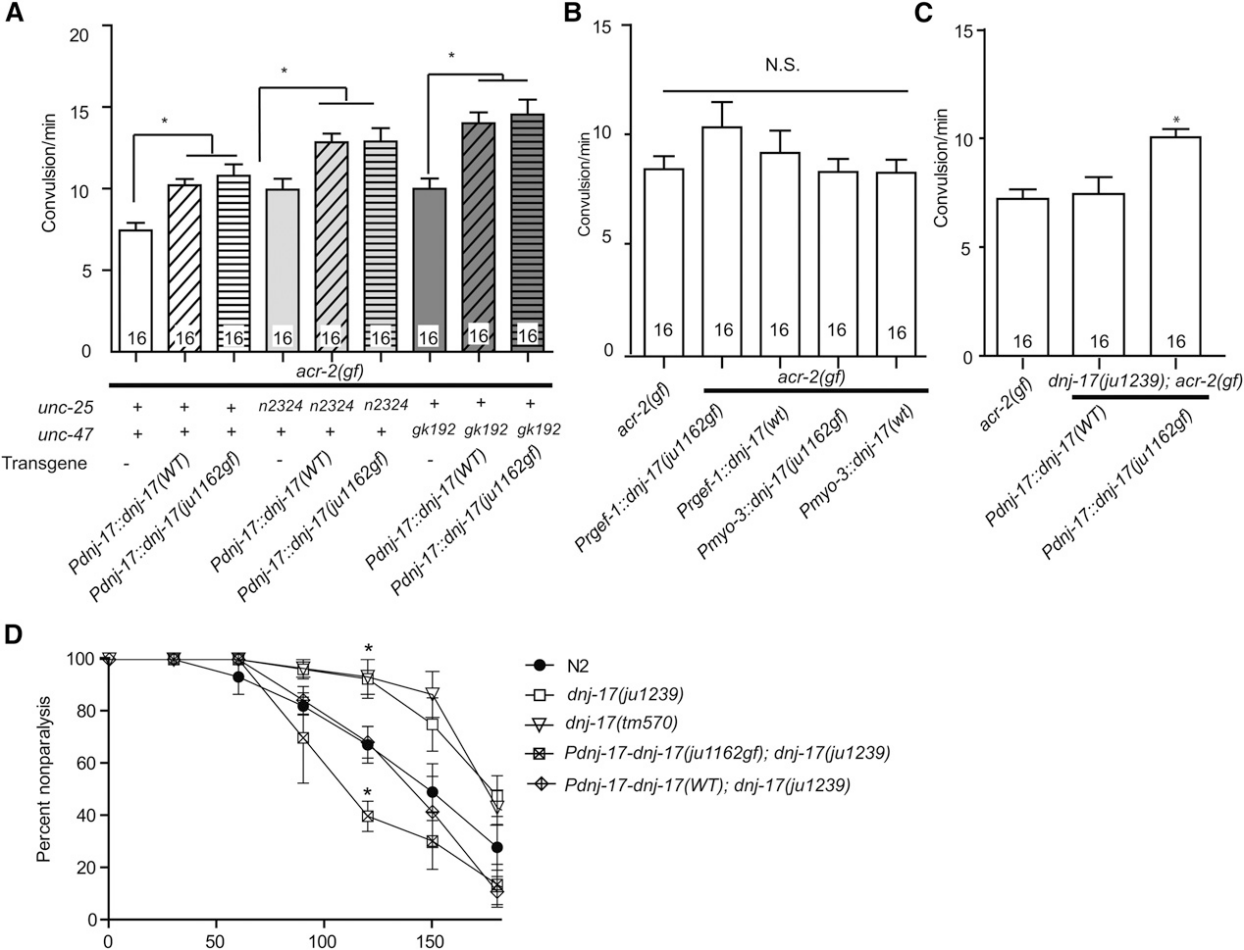
Single-copy expression of *dnj-17(ju1162gf)* is sufficient to cause increase in convulsion frequency. (A−C) Quantification of convulsion frequency. (A) Overexpression of *dnj-17(+)* or *dnj-17(ju1162gf)* by high-copy extrachromosomal arrays causes increase of *acr-2(gf)* convulsion frequency. (B) Neuron- or muscle-specific overexpression of *dnj-17* does not affect convulsion frequency. (C) Single-copy expression of *dnj-17(ju1162gf)*, but not *dnj-17(+)*, causes increase in convulsion frequency. Statistics, one way ANOVA followed by Bonferroni’s *post hoc* test. * *P* < 0.05. Error bars indicate SEM. Numbers in the column indicate sample sizes. (D) Aldicarb resistance of *dnj-17(ju1239)* null is suppressed by expression of single-copy wild-type *dnj-17. dnj-17(ju1239)* animals with single-copy expression of *dnj-17(ju1162gf)* showed mildly increased sensitivity to aldicarb. Statistics, two way ANOVA. * *P* < 0.05 compared to N2 at the given time point.

We next examined the nature of DNJ-17(N77K) using transgenic overexpression. Overexpression of wild-type *dnj-17* by genomic sequence of *dnj-17* including 0.9 kb upstream promoter region caused increase of *acr-2(gf)* convulsion frequency (Figure 4A). This transgene also enhanced convulsion frequency in *unc-47(0)* mutant background, suggesting that the increase in convulsion by overexpression of *dnj-17* is independent of the effect caused by defects in GABAergic transmission. Interestingly, this enhanced effect was also observed by overexpression of *dnj-17(ju1162gf)*. ACR-2 is expressed specifically in neurons (Jospin *et al.* 2009). However, overexpression of *dnj-17* wild type or *ju1162(gf)* using the *Prgef-1* pan-neuronal promoter did not affect convulsion frequency of *acr-2(gf)* (Figure 4B). Also, overexpression in muscles using *Pmyo-3* promoter did not affect *acr-2(gf)* convulsion frequency (Figure 4B). These results suggest that the effect of *dnj-17* on convulsion frequency likely requires its expression in multiple tissues.

To precisely compare the effect of *dnj-17(ju1162gf)* to wild-type *dnj-17*, we generated a single-copy insertion transgene expressing full-length genomic *dnj-17(ju1162gf)* or *dnj-17(+)* on chromosome II. Animals with *Pdnj-17-dnj-17(ju1162gf)* expressed from a single-copy transgene showed overall normal locomotion, growth speed, and brood size. We found that *Pdnj-17-dnj-17(ju1162gf);acr-2(gf)* increased convulsion compared to *acr-2(gf)* single mutants, whereas *Pdnj-17-dnj-17(+)* single-copy expression did not (Figure 4C), consistent with *dnj-17(ju1162gf)* acting semidominantly in *unc-25(e156)* background (Figure 2D). These results suggest that the DNJ-17(N77K) mutation has higher activity than wild-type DNJ-17, implying that the increase in convulsion by overexpression of wild-type *dnj-17* is caused by excess levels of the protein.

### dnj-17 activity affects the response to aldicarb

To further assess the effect of *dnj-17* mutations on neurotransmission at the neuromuscular junction, we examined the sensitivity of the mutant animals to an acetylcholine esterase inhibitor aldicarb. *dnj-17(ju1239)* null animals showed mild resistance to aldicarb, which was rescued by single-copy insertion of *dnj-17(+)*, implying that *dnj-17* affects cholinergic transmission at the neuromuscular junction (Figure 4D). Expression of *dnj-17(ju1162gf)* caused mildly increased sensitivity to aldicarb, reaching statistical significance at one time point, consistent with this allele being a hypermorph mutation. These results raise a possibility that the function of *dnj-17* is required for folding and/or function of proteins in multiple tissues that are involved in cholinergic transmission. Overexpression of wild-type DNJ-17 may also lead to a high level of cholinergic transmission by contributing to folding of the proteins in the pathway.

### Perspectives

Other mechanisms might account for the effects of *dnj-17(ju1162)*. The N77K mutation may make DNJ-17 protein prone to form aggregates. DnaJ/Hsp40 proteins bind to misfolded proteins and bring them to Hsp70 (Cheetham and Caplan 1998). The N77K mutation could alter the kinetics for DNJ-17 to detach from the protein(s) it binds to, and prevent the misfolded protein from being degraded, resulting in accumulation of misfolded proteins that cause cellular stress. Such cellular stress could alter neuronal and muscular functions. Another possibility is that the mutation disrupts certain cellular functions. Recently it was reported that an Asn to Ser mutation in the DnaJ domain of human DNAJC13 was found in a family with Parkinson disease, where the disease was transmitted in an autosomal-dominant manner (Vilariño-Güell *et al.* 2014). The mutant protein exhibited a toxic gain-of-function activity affecting endosomal transport. The N77K mutation in *C. elegans* DNJ-17 may affect similar cellular functions such as endocytosis and subcellular trafficking, thus disrupting the coordination of the motor neuron circuit.

*E. coli* has only one gene coding DnaJ/Hsp40, whereas animals typically express multiple DnaJ family members. The DnaJ protein in *E. coli* has been well characterized, but functions of individual DnaJ/Hsp40 family proteins in animals remain largely unknown. DNAJA5, the closest human homolog of DNJ-17, shows enhanced expression in the brain (Chen *et al.* 2004) which suggests neuron-specific roles, but its substrates and functions are yet to be characterized. Studies of DnaJ/Hsp40 in animals may lead to further understanding of the physiological mechanisms of protein homeostasis in neurodegenerative diseases.

## Supplementary Material

Supplemental Material
