# Supplementary material for: Altered Function of the DnaJ Family Cochaperone DNJ-17 Modulates Locomotor Circuit Activity in a Caenorhabditis elegans Seizure Model
Source: G3 (Bethesda). 2016 May 16;6(7):2165–71. doi: 10.1534/g3.116.028928 (PMC4938669; doi:10.1534/g3.116.028928)
Supplement: Supplemental Material [file supp_6_7_2165__index.html]

Altered Function of the DnaJ Family Cochaperone DNJ-17 Modulates Locomotor Circuit Activity in a Caenorhabditis elegans Seizure Model — Supplemental Material 

# Altered Function of the DnaJ Family Cochaperone DNJ-17 Modulates Locomotor Circuit Activity in a *Caenorhabditis elegans* Seizure Model

## Supplemental Material for Takayanagi-Kiya and Jin, 2016

**Files in this Data Supplement:**

- Table S1 - List of strains and transgenes used in the study. (.xlsx, 13 KB)
- Table S2 - List of constructs used in the study. (.xlsx, 11 KB)
